# Impact of Natalizumab Treatment on Fatigue, Mood, and Aspects of Cognition in Relapsing–Remitting Multiple Sclerosis

**DOI:** 10.3389/fneur.2015.00097

**Published:** 2015-05-11

**Authors:** Annett Kunkel, Martin Fischer, Judith Faiss, Doreen Dähne, Wolfgang Köhler, Jürgen H. Faiss

**Affiliations:** ^1^Department of Neurology, Asklepios Fachklinikum Teupitz, Teupitz, Germany; ^2^Department of Neurology, Fachkrankenhaus Hubertusburg, Wermsdorf, Germany

**Keywords:** multiple sclerosis, cognition, fatigue, depression, natalizumab

## Abstract

**Background/objective:**

Fatigue, cognitive, and affective disorders are relevant symptoms in multiple sclerosis (MS). The treatment with Natalizumab has a positive effect on physical disabilities in patients with relapsing–remitting MS (RRMS). Some studies describe improvements in cognition and fatigue over 1 year of treatment. Only little is known about longer treatment effects especially on fatigue, and also on cognition and mood. Therefore, the present retrospective open label observational study investigates the effect of Natalizumab on fatigue, attention, and depression over a treatment period of 2 years.

**Methods:**

About 51 RRMS patients who were treated with Natalizumab (male = 11, female = 40; mean age: 33. 9 ± 9. 1 years) were included. The neuropsychological assessment consisted of different tests of attention (TAP: alertness, divided attention, flexibility, SDMT, PASAT), fatigue (WEIMuS, FSMC), and depression (CES-D). The assessments occurred immediately before the first administration of Natalizumab, after 1 and 2 years of treatment.

**Results:**

Significant improvements were found in aspects of attention and depression from baseline to follow-up 1 [alertness: reaction time (RT) cued, *p* < 0.05; divided attention: visual RT, *p* < 0.05; SDMT: *p* = 0.05; CES-D: *p* < 0.05] and from baseline to follow-up 2 (divided attention: visual RT: *p* < 0.001; errors: *p* < 0.01, omissions: *p* < 0.05; flexibility: RT, *p* < 0.05; SDMT: *p* < 0.01; CES-D: *p* < 0.05). No significant changes were detected in fatigue, probably because of the small sample size, especially in the second year of treatment (WEIMuS: *N* = 16, FSMC: *N* = 8).

**Conclusion:**

The results show a positive effect of Natalizumab on attention in patients with RRMS, and for the first time, also in depression after 2 years of observation, and support the efficacy of the treatment over 2 years. More research is needed for fatigue.

## Introduction

Multiple sclerosis (MS) is a chronic inflammatory demyelinating disease of the central nervous system, which causes myelin destruction and axonal loss in the brain and spinal cord, and leads to different visible and invisible symptoms, such as MS-related fatigue, cognitive dysfunctions, and depression (1, 2). Fatigue affects up to 90% of MS patients in all disease stages (3, 4), has significant socioeconomic consequences, and is a relevant factor of diminished quality of life among patients with MS (5). Different biological and psychological models exist regarding the multifactorial etiology of fatigue (6). Inflammation, demyelination, and also behavioral variables such as anxiety, depression, and reduced activity are associated with fatigue (7).

In addition, cognitive dysfunctions are common in MS (8) and can appear in all disease stages (9–11). The association to fatigue is unclear. Whereas some studies describe an association between fatigue and cognitive impairment (12, 13), in other studies no correlation was found (14, 15). The prevalence rates vary between 43 and 70% depending on the research setting, the characteristics of the clinical sample, and the used assessment tools (16). Most impaired cognitive domains in MS are information processing speed and complex attention, verbal and non-verbal memory, and executive functions, as well as visual spatial functions. Intelligence, language, and semantic memory are mostly preserved (15, 17, 18).

Depression in MS has a prevalence rate up to 50% (19, 20), and is associated with fatigue as well as cognitive functions (1, 21). The differentiation between fatigue and depression is often difficult and symptoms of depression can be mistaken as fatigue.

Different studies describe a beneficial effect of immunmodulatory treatments on cognitive functions by containing the development of new cerebral lesions or reducing brain atrophy (22–24). Less clearly are the effects of immunmodulatory therapies on fatigue and depression. Metz et al. (25) report a more beneficial effect of glatirameracetat than β interferon on fatigue after 6 months of treatment. Other studies found no relationship between depression and treatment with interferons (26, 27). One study reported decreased quality of life, worsened fatigue, and depression under treatment with β interferon (28).

Natalizumab (NTZ) is a humanized monoclonal antibody, and is used as monotherapy in relapsing–remitting MS (RRMS) in severe courses. NTZ has positive effects on physical disabilities, in reducing the relapse rate and magnetic resonance imaging (MRI) detectable disease activity (29, 30). Past studies investigated the effect of NTZ on cognition and partially also on depression and fatigue over the observational periods of 6 months to 1 year (31–35). Putzki et al. (36) encompassed fatigue and depressive symptoms at baseline immediately before the treatment with NTZ and 6 months later. About 46% of the 42 treated patients decreased in the Modified Fatigue Impact Scale [MFIS, (37)] and 39% showed improvements in the Fatigue Severity Scale [FSS, (38)]. No changes were detected in the Beck′s Depression Inventory [BDI, (39)] over the observational period. Another multicenter study (34) with 195 RRMS patients found significant improvements in fatigue after 12 months of treatment with NTZ. Fatigue was measured with the Fatigue Scale for Motor and Cognitive Functions [FSMC, (40)]. All of the secondary outcomes improved over time, including quality of life, sleepiness, depression, cognition, and the degree of disability.

Little is known about the treatment effects of NTZ for longer treatment periods and the effect on specific cognitive domains. Iaffaldano et al. (41) found improvements in fatigue and cognition after 1 year of treatment and partially for fatigue also after 2 years of NTZ treatment. Fatigue was measured with the FSS (38) and improved from initially 45% of patients with fatigue to 29% after 1 year of treatment. This effect remained stable in the second year of treatment. Cognitive impairment, measured by Rao’s Brief Repeatable Battery (BRB) and Stroop Test (42), improved significantly only in the first year of treatment (29% to 19% decrease); in the second year, the effect was not statistical significant.

Against the background of the results in the literature and in comparison and addition to the hitherto published data, the aim of the present retrospective open label observational study was the investigation of the effect of NTZ on fatigue, different aspects of cognition, and depression over a treatment period of 1 and 2 years.

## Materials and Methods

### Study design and sample

The present retrospective open label observational study was conducted at two departments of neurology (Teupitz, Wermsdorf) in Germany. Both are accredited MS centers from the German Multiple Sclerosis Association (DMSG). All patients were explained in detail prior to the first application of NTZ through competent MS nurses and the attending physicians; and provided their oral and written informed consent for all procedures; the participation and the accompanying required comprehensive clinical assessment. All used data were made anonymous and were transferred without names, addresses, and date of birth in an evaluation file. No study code was used. Consequently, a subsequent assignment from the data to a special patient record is not possible.

Data of 51 (Teupitz: *N* = 33, Wermsdorf, *N* = 18) patients with a relapsing remitting course of MS (43), who were treated monthly with a constant dose of 300 mg NTZ according to the german pharmaceutical indications over the course of 1 year, were included. Thirty one of these patients continued the application with NTZ for a second year of treatment. The remaining 20 cases completed the second treatment year after time of statistical analysis in the course of the ongoing year. The detailed description of the sample is shown in Table 1. Before the treatment with NTZ, most patients had applied other treatment options. Twenty seven patients received Interferon-β1a, 24 received Interferon-β1b, 12 patients were treated with Glatiramer acetate, and 4 patients got Mitoxantron.

**Table 1:** **Description of the sample**.

	Baseline	Year 1	Year 2
*N*	51	51	31
Age	33.9 ± 9.1^a^		
Gender (male/female)	11/40	11/40	7/24
Disease duration (years)	5.3 ± 4.8^a^		
EDSS^b^	4.0 ± 1.6^a^	3.8 ± 1.7	3.9 ± 1.7
Number of prior medications	1.3 ± 0.8		
Time to follow-up (years)		0.9 ± 0.2	2.0 ± 0.4
Depression-index (CES-D)	18.5 ± 10.1	16.6 ± 10.2	16.6 ± 8.9
	Cut-off > 22 = 15		
Fatigue-index (FSMC)	65.2 ± 17.5	65.8 ± 20.5	63.4 ± 20.5
	Cut-off ≥ 43 = 9		
Fatigue-index (WEIMuS)	32.7 ± 15.7	29.6 ± 18.3	29.6 ± 20.2
	cut-off ≥ 32 = 15		

*^a^Mean ± SD*.

*^b^Expanded Disability Status Scale (44)*.

*Prior medications: interferon-β1a, interferon-β1b, glatiramer acetate, Mitoxantron*.

*WEIMuS, Würzburg Fatigue Inventory for MS (45); FSMC, Fatigue Scale for Motor and Cognitive Functions (40); CES-D, Center for Epidemiological Studies-Depression Scale (46, 47)*.

### Clinical and cognitive assessments

Prior to the first application of NTZ, every patient received a standardized comprehensive clinical assessment, consisting of MRI, chest X-ray, gynecological, respectively, urologic check-up, inspection of the skin, determination of visual acuity, sonography of the epigastrum, different blood tests, JVC-antibody test, and the neurological examination with assessment of the degree of disability measured by EDSS (44). The treatment with NTZ included yearly follow-up examinations of MRI, blood test, cognitive assessment, JVC-antibody test, and neurological examination (EDSS).

Furthermore, all patients obtained an annually comprehensive cognitive testing for detecting changes in cognition during the treatment of NTZ. The cognitive assessment was performed by trained psychologists according to standard procedures. All cognitive tests were performed immediately before the first infusion of NTZ, after 1 and 2 years of application with a fixed test battery. All examinations at each measurement point start with the assessment of fatigue and depression. For the evaluation of fatigue, the Fatigue Scale for Motor and Cognitive Functions [FSMC; (40)] and the Würzburg Fatigue Inventory for MS [WEIMuS; (45)] were used. The FSMC consists of 20 items and is a five-stage rating scale without time limit for evaluation. The cut-off for fatigue is ≥43. On the other hand, the WEIMuS estimates fatigue in the course of the last week. This questionnaire consists of 17 items and has also a 5-stage rating scale with a cut-off for fatigue of ≥32. For the assessment of depressive symptoms, the 20 items of the Center for Epidemiological Studies-Depression Scale [CES-D; (46, 47)] were used. The cut-off for a clinical relevant depression is >22. This cut-off achieves a high sensitivity (82–84%) and specificity (47).

In everyday clinical practice, the neuropsychological examination included the computerized Attention Test Battery [TAP, Version 2.2; (48)], with the subtests alertness (responsiveness), divided attention and flexibility, as well as the Symbol Digit Modalities Test [SDMT; (49)], for the assessment of different aspects of attention and information processing speed. Additionally, verbal memory was tested using the Auditory Verbal Learning test (50) and non-verbal memory using the “Diagnosticum fuer Cerebralschaedigung” [DCS; (51)]. Visuospatial abilities were assessed by the Rey–Osterrieth Complex Figure Test (ROCF) using the Taylor Scoring System (52). A Word Fluency Test was used for a screening of executive functions with the subtests animals, S-words, and alternating G- and R-words (53). In addition, a recognition vocabulary test was used to assess premorbid aspects of intelligence [German Vocabulary Scale, WST; (54)]. All cognitive tests were given in a fixed order: SDMT, AVLT (learning, interference), ROCF, three subtests of the TAP, AVLT (recall and recognition), DCS, fluency, WST.

### Statistical analysis

Statistical analyses were performed with the Statistical Package for the Social Sciences (SPSS Inc., Chicago, IL, USA). Only the data concerning fatigue, depression, and attention were analyzed. Tests for memory and executive functions were not analyzed because of the missing control group, against the background of learning effects. Variables were reported by using descriptive statistics. Differences from baseline to follow-up measurement points (year 1, year 2) were evaluated using the *t*-test for paired samples and the Wilcoxon Rank test. The statistical analysis for 2 years is based only on those patients who remained in the study the whole time (*N* = 31), because in the utilized paired sample *t*-test only those cases were included for which data for both time points were available. The *p*-value <0.05 was considered as statistical significant on one-sided testing. Not all tests could be given at all measurement points because of limited physical and cognitive skills. The following 10 test-parameters were used for the statistical analysis:

1. Attention Test Battery
  Alertness:
  median reaction time (AL RT), measured in milliseconds (ms).
  median reaction time cued (AL RT cued) in ms (responsiviness).
  Divided Attention:
  median divided attention reaction time visual (Divid Att RT vis) in ms.
  median divided attention reaction time auditory (Divid Att RT aud) in ms.
  divided attention errors (Divid Att errors).
  divided attention omissions (Divid Att omissions).
  Flexibility:
  median flexibility reaction time (Flex RT) in ms.
  flexibility errors (Flex errors).
  flexibility performance index (Flex perfom index).
2. SDMT
  number of correct answers.

For the assessment of fatigue and depression with the WEIMuS, FSMC, and CES-D, the total values were used for the statistical analysis.

## Results

The statistical analysis was subdivided into three parts. In the first part, the data of the total sample were analyzed. The second and third part describes the data of the subsamples of patients with fatigue and depression, respectively.

### Total sample

Fifty-one patients (11 men, 40 women) with an age between 19 and 51 years (Median: 33.9 ± 9.1) were treated with NTZ for 1 year. The mean disease duration was 5.3 ± 4.8 years (minimum: 0.34 years, maximum: 17.65 years). The average number of MS medications prescribed prior to NTZ was 1.3 ± 0.8. The time between baseline and first follow-up assessment after 1 year of treatment was in mean 0.9 ± 0.2 years. About 31 of these patients (7 men, 24 women) were treated for a second year with NTZ (mean time to follow-up from baseline to year 2 was 2.03 ± 0.4 years). No significant changes were found in the degree of disability, measured by EDSS from baseline (EDSS = 3.99 ± 1.55) to year 1 (EDSS = 3.77 ± 1.73; *p* = 0.06) and from baseline (EDSS = 4.13 ± 1.65) to year 2 (EDSS = 3.90 ± 1.68; *p* = 0.19) for the total sample.

At baseline, the mean fatigue scores (WEIMuS, FSMC) were 30.75 ± 14.76 and 64.71 ± 21.19, respectively, as shown in Table 2, ranging from 0 to 64 points in the WEIMuS and 31 to 93 points in the FSMC. From baseline to year 1, as well as to year 2, no significant changes were observed (Table 2). In the second year of treatment, the WEIMuS ranged between 0 and 66 points and the FSMC ranged between 20 and 91 points.

**Table 2:** **Changes in fatigue and depression over the total sample and both years of observation**.

	First year of treatment		Second year of treatment	
	Baseline *N* = 51	Year 1 (p*) *N* = 51	Baseline *N* = 31	Year 2 (p*) *N* = 31
WEIMuS	30.75 ± 14.76^a^	29.06 ± 18.67 (0.19)	30.94 ± 14.65	28.88 ± 21.18 (0.27)
FSMC	64.71 ± 21.19^a^	64.29 ± 23.85 (0.47)	69.50 ± 10.66	73.00 ± 12.35 (0.31)
CES-D	18.96 ± 10.07^a^	16.83 ± 10.21 (0.06)	19.16 ± 8.97	16.16 ± 8.95 (0.07)

*WEIMuS, Würzburg Fatigue Inventory for MS; FSMC, Fatigue Scale for Motor and Cognitive Functions; CES-D, Center for Epidemiological Studies-Depression Scale (german variant, longform)*.

*^a^Mean ± SD*.

*p-value following paired sample *t*-test.

In depression, measured by the CES-D, only a statistical trend for significant changes over both time points were observed in the total sample (Table 2).

The analysis of cognitive parameters (Table 3) showed significant changes after 1 year of treatment in 3 out of 10 parameters, and 5 out of 10 parameters in the second year of treatment. Significant changes were evident in the subtests alertness [responsiveness (alertness): *p* < 0.05], and divided attention of the TAP (median reaction time visual: *p* < 0.05), and the SDMT (*p* < 0.05). After the second year of treatment, the effect in the divided attention was stable for the visual reaction time (*p* < 0.001) and extended for the error rate (*p* < 0.01) and number of omissions (*p* = 0.05). Furthermore, the reaction time, as part of cognitive flexibility, significantly changed from baseline to year 2 (*p* = 0.05). Above that, the number of correct answers in the SDMT was significant better after the second year of treatment in comparison to baseline (*p* < 0.01).

**Table 3:** **Changes in different aspects of attention over 2 years of treatment with NTZ**.

	First year of treatment		Second year of treatment	
	Baseline *N* = 51	Year 1 (p*) *N* = 51	Baseline *N* = 31	Year 2 (p*) *N* = 31
AL RT^a^	279.05±73.11	268.49±54.30 (0.13)	294.23±86.15	283.39±65.65 (0.22)
AL RT cued^a^	277.16±84.01	**256.75 ***±*** 37.25 (0.02)**	289.23±102.96	269.35±48.74 (0.11)
Divid Att RT vis^a^	884.24±182.45	**844.22 ***±*** 137.60 (0.02)**	912.29±214.89	**832.66 ***±*** 165.45 (0.00)**
Divid Att RT aud^a^	612.00±91.04	614.04±89.71 (0.42)	626.50±85.56	623.39±91.33 (0.41)
Divid Att errors	2.36±4.06	1.55±2.85 (0.11)	2.75±4.97	**1.14 ***±*** 2.01 (0.01)**
Divid Att omissions	2.06±0.96	1.72±1.49 (0.12)	2.25±1.95	**1.64 ***±*** 1.54 (0.05)**
Flex RT^a^	931.07±778.20	769.97±255.33 (0.07)	1116.31±1040.21	**738.78 ***±*** 260.58 (0.05)**
Flex errors	3.94±6.80	2.24±2.07 (0.25)	4.83±8.71	1.50±1.46 (0.10)
Flex Perform Index	5.09±6.56	4.18±8.56 (0.39)	4.95±5.11	8.36±7.90 (0.07)
SDMT	49.06±10.01	**51.88 ***±*** 7.88 (0.02)**	46.67±9.30	**53.08 ± 7.53 (0.01)**

*^a^In milliseconds (ms)*.

*All values are presented as means ± SD*.

*AL RT, alertness, reaction time; AL RT cued, alertness reaction time cued; Divid Att RT vis, divided attention reaction time visual; Divid Att RT aud, divided attention reaction time auditory; Divid Att errors, divided attention errors; Divid Att ommisions, divided attention omission; Flex RT, flexibility reaction time; Flex errors, flexibility errors; Flex Perfom Index, flexibility performance index; SDMT, symbol digit modalities test*.

*p-value following paired sample *t*-test.

*The bold font indicates the statistical significant results*.

### Patients with fatigue

At baseline, 28 patients (54.90%) of the total sample reported a fatigue syndrome. After the first year of treatment with NTZ, again, 28 patients (54.90%) of the sample complaint about fatigue, and after 2 years of treatment still 19 patients (61.29%) reported a fatigue syndrome. Therefore, no significant changes in fatigue were observed over the three measurement points (Table 4).

**Table 4:** **Analysis of cognitive parameters from patients with fatigue at baseline**.

Patients with fatigue	First year of treatment		Second year of treatment	
	Baseline *N* = 28	Year 1 (p*) *N* = 28	Baseline *N* = 16	Year 2 (p*) *N* = 16
AL RT^a^	277.39±73.20	271.16±65.55 (0.33)	297.75±87.16	280.00±62.01 (0.14)
AL RT cued^a^	279.61±87.85	**255.36 ***±*** 40.29 (0.04)**	295.25±110.03	266.19±50.43 (0.12)
Divid Att RT vis^a^	856.41±152.23	834.00±135.99 (0.16)	900.47±186.05	**811.20 ***±*** 187.30 (0.03)**
Divid Att RT aud^a^	619.26±93.08	627.22±89.85 (0.31)	630.33±92.93	632.00±82.70 (0.46)
Divid Att errors	2.59±4.19	2.26±3.54 (0.46)	2.80±5.26	1.40±2.44 (0.07)
Divid Att omissions	2.11±2.08	1.81±1.54 (0.22)	2.60±2.13	**1.67 ***±*** 1.76 (0.02)**
Flex RT^a^	763.25±219.31	778.75±232.57 (0.36)	869.57±269.18	**744.00 ***±*** 294.57 (0.04)**
Flex errors	2.00±3.26	2.69±2.44 (0.17)	2.43±4.72	1.00±0.82 (0.34)
Flex Perform Index	6.97±6.45	**2.17 ***±*** 10.13 (0.03)**	6.22±6.52	8.06±9.47 (0.14)
SDMT	48.91±9.85	51.45±7.85 (0.05)	47.00±9.62	52.75±9.16 (0.05)
WEIMuS^b^	42.19±7.90	41.31±13.18 (0.37)	43.50±8.14	39.50±18.22 (0.19)
FSMC^b^	70.17±17.01	64.67±26.10 (0.14)	69.50±10.66	73.00±12.35 (0.31)
CES-D	22.04±8.24	19.19±6.63 (0.06)	22.15±8.52	17.46±7.11 (0.06)

*^a^In milliseconds (ms)*.

*All values are presented as means ± SD*.

*^b^WEIMuS and FSMC: first year: *N* = 16, second year: *N* = 8*.

*AL RT, alertness, reaction time; AL RT cued, alertness reaction time cued; Divid Att RT vis, divided attention reaction time visual; Divid Att RT aud, divided attention reaction time auditory; Divid Att errors, divided attention errors; Divid Att ommisions, divided attention omission; Flex RT, flexibility reaction time; Flex errors, flexibility errors; Flex Perfom Index, flexibility performance index; SDMT, symbol digit modalities test; WEIMuS, Würzburg Fatigue Inventory for MS; FSMC, fatigue Scale for Motor and Cognitive Functions; CES-D, Center for Epidemiological Studies-Depression Scale*.

*p-value following paired sample *t*-test.

*The bold font indicates the statistical significant results*.

The subgroup of patients with fatigue did not differ in age, disease duration, EDSS, and cognitive performance from the other patients (results of the independent *t*-tests are not shown, because they are not significant).

Table 4 shows the changes over time in the attention parameters in the subgroup of patients with fatigue. After the first year of treatment, 2 out of 10 cognitive parameters were significantly changed (one trend toward significance in SDMT), and in the second year 3 out of 10 parameters showed significant changes (two trends). From baseline to year 1, patients with fatigue reached significant improvements in the subtests alertness (*p* < 0.05) and flexibility (*p* < 0.05). The SDMT showed a trend toward significance (*p* = 0.05). In year 2, different parameters of divided attention (reaction time visual, *p* < 0.05; omissions, *p* < 0.05) and flexibility (reaction time, *p* = 0.05) significantly improved. The SDMT also showed a trend toward significance.

Furthermore, changes in the value of depression were observed. At baseline, the mean CES-D (22.04 ± 8.24) reached the cut-off for a clinical relevant depression. After 1 year of treatment as well as after 2 years, the CES-D value decreased in mean to 19.19 in year 1 and 17.46 in year 2. Both improvements showed a trend toward significance.

### Patients with depression

In contrast to the total sample, a clear improvement in the CES-D value was evident over the observational period. At baseline, 15 patients (29.41%) reported a CES-D value that suggests a clinically relevant depression, in contrast to 21.56% (11 patients) after 1 year and 22.58% (7 patients) after 2 years of NTZ treatment. The CES-D values decreased significantly from baseline to year 1 [*t* = 2.17 (14), *p* < 0.05] and from baseline to year 2 [*t* = 2.81 (8), *p* < 0.05]. No significant changes were detected between year 1 and year 2 [*t* = 1.45 (8), *p* = 0.18].

Changes in cognitive parameters, as shown in Table 5 were detected similar to the total sample in the divided attention (baseline to year 1: omissions: *p* < 0.05; baseline to year 2: visual reaction time: *p* < 0.05) and flexibility (baseline to year 2: flexibility reaction time: *p* < 0.05). In the first year, one cognitive parameter improved, and in the second year two parameters.

**Table 5:** **Analysis of cognitive parameters from patients with depression at baseline**.

Patients with depression	First year of treatment		Second year of treatment	
	Baseline *N* = 15	Year 1 (p*) *N* = 15	Baseline *N* = 9	Year 2 (p*) *N* = 9
AL RT^a^	265.17±41.55	284.80±63.93 (0.07)	275.39±42.51	282.78±47.98 (0.32)
AL RT cued^a^	266.47±55.03	268.27±33.95 (0.45)	270.67±63.34	272.89±35.06 (0.45)
Divid Att RT vis^a^	859.43±161.07	827.73±95.46 (0.15)	900.06±194.25	**833.67 ***±*** 140.78 (0.02)**
Divid Att RT aud^a^	604.93±77.83	614.20±80.100 (0.32)	613.00±55.94	611.67±58.51 (0.48)
Divid Att errors	1.40±1.88	1.00±1.06 (0.14)	1.44±2.35	0.78±1.64 (0.07)
Divid Att omissions	2.07±1.98	**1.13 ***±*** 1.12 (0.03)**	2.89±2.03	1.44±1.94 (0.04)
Flex RT^a^	867.81±255.95	820.04±227.88 (0.17)	964.50±305.81	**762.14 ***±*** 260.07 (0.03)**
Flex errors	2.85±3.51	2.69±2.52 (0.48)	3.14±4.67	1.00±0.82 (0.11)
Flex Perform Index	2.75±4.38	− 0.31±9.11 (0.12)	4.48±5.35	5.42±6.37 (0.21)
SDMT	51.00±4.24	52.50±2.12 (0.25)		
WEIMuS	40.73±8.75	39.18±15.13 (0.35)	43.20±10.37	35.40±19.34 (0.27)
**CES-D**	**30.13 ***±*** 6.26**	**24.00 ***±*** 11.77 (0.02)**	**28.67 ***±*** 4.35**	**18.44 ***±*** 9.98 (0.01)**

*^a^In milliseconds (ms)*.

*All values are presented as means ± SD*.

*AL RT, alertness, reaction time; AL RT cued, alertness reaction time cued; Divid Att RT vis, divided attention reaction time visual; Divid Att RT aud, divided attention reaction time auditory; divid Att errors, divided attention errors; Divid Att ommisions, divided attention omission; Flex RT, flexibility reaction time; Flex errors, flexibility errors; Flex Perfom Index, flexibility performance index; SDMT, symbol digit modalities test; WEIMuS, Würzburg Fatigue Inventory for MS*.

*p-value following paired sample *t*-test.

*The bold font indicates the statistical significant results*.

Concerning fatigue, no significant changes were observed in the observational period. After the first year of NTZ treatment, the WEIMuS-score was almost identical (Table 5). In the second year, the score decreased about some points, but without statistical significance.

## Discussion

In this retrospective open label observational study, cognitive performance, depression, and fatigue of 51 RRMS patients treated with NTZ over 1 year, and 31 of them treated over 2 years with NTZ were investigated.

Looking at the total sample, the patients cognitively improved in different aspects of attention, namely responsiveness (alertness), information processing speed, and divided attention (visual reaction time) after 1 year of treatment with NTZ. After 2 years of treatment, additional effects were evident in errors and omissions in divided attention and the flexibility reaction time.

About 55% of the patients suffered from fatigue at baseline. This value even increased to 61% in the second treatment year. Although the fatigue itself was not affected by the treatment, patients suffering from fatigue showed improvements in responsiveness, divided attention, information processing speed, and flexibility after the first and the second year of treatment.

The data suggests that NTZ may have a positive effect on depression in patients with RRMS. The CES-D values of the 29% patients with a baseline depression decreased so that after the first and second year of treatment, only 22% suffered from depression.

The present results are partially comparable to the hitherto published data. The main differences are the lack of improvement in fatigue in the present sample in comparison to the data in the literature and the improved depression. Three published studies have longitudinally assessed the effect of NTZ especially on fatigue as primary outcome variable (34, 36, 41).

Putzki et al. (36) described a contrary effect in comparison to the present data. In their study, an improvement in fatigue but not in depression was detected, however, over a shorter observational period of 6 months. In addition, other assessment tools (MFIS, FSS, BDI) were used, so that the results are not completely comparable to the present data. Especially, the BDI encompasses other aspects of depression as the CES-D. For patients with MS, the CES-D seems to be more appropriate. Furthermore, the different treatment periods with NTZ can be a reason for the different results. Against the background of learning effects, longer time periods for retesting are recommended.

Svenningson et al. (34) found in a considerably greater sample (195 patients), an improvement in fatigue over an observational period of 1 year. In this study comparable to the present study, the FSMC was used for assessing fatigue and the CES-D for evaluation of depression. Patients with a higher fatigue score and a lower depression score at baseline showed a stronger improvement after 12 months of treatment. Fatigue reduced from severe to moderate, according to the FSMC, a well reviewed fatigue scale in MS (55). Secondly and comparable to the present data, an improvement in depression was observed. The CES-D score improved from initially 18.3 before treatment to 14.2 after 12 months of treatment. Such a trend was also observed in the present study (decrease from 18.96 at baseline to 16.83 year 1).

In the study from Iffaldano et al. (41), 100 patients with RRMS were treated with NTZ over 2 years. As mentioned above, cognitive parameters were assessed by the BRB and Stroop paradigma, fatigue was measured with the FSS. Fatigue reduced from initially 45 (FSS = 4.01 ± 1.63) to 29% (FSS = 3.61 ± 1.56) after 1 year of treatment and from 52.8 [28 (53) patients] to 34% [18 (53) patients] in the second year. Regarding the cognition at baseline, 29% of patients were classified as cognitive impaired and failed at least in three tests of the BRB and Stroop test. After 1 year of treatment, the number of cognitive impaired patients decreased to 19%, and in the subgroup of patients with 2 years of treatment from initially 22.6 to 17%, which was statistical significant. In contrast to the present data, no effect was found in depression, measured again with the BDI.

Changes in fatigue as well as in cognition during 12 weeks of treatment were described also by Wilken et al. (35). They used three different fatigue assessment tools (MFIS, FSS, visual analog scale). The observed effect improved or remained stable up to 48 weeks after initiation of NTZ treatment.

The reason for the different fatigue results between the present data and previously published data can be the small sample sizes, especially in the second year of treatment in the present study as well as the different assessment periods of the used questionnaires. The WEIMuS evaluates the behavior only of the last week, whereas the FSMC use an overall assessment without time limit. However, in the literature, predominantly other assessment tools were used (FSS, MFIS) with again other assessment periods. The MFIS focuses on the last 4 weeks, the FSS asks for fatigue in general. Furthermore, there are differences in the content of the questionnaires. Whereas, the FSS focus on fatigue in general, the MFIS and FSMC ask for fatigue more specific, and may evaluate cognitive and physical fatigue separately. Therefore, the comparability of the published results is limited. Recently, the FSMC is the recommended questionnaire for assessment of fatigue in MS (55).

Other studies with different observational periods describe comparable to the present data, mostly a positive effect of NTZ on different aspects of cognition. Lang et al. (32) found significant improvements in cognition after 6 months of treatment with NTZ in a sample of 29 patients with RRMS. Mainly improvements were detected in verbal and non-verbal memory, alertness, quality of life, depression, and fatigue. A total of 15 (30) assessed parameters improved over time, 15 remained equal. These results are comparable with another sample of 40 patients with RRMS (31), treated with NTZ over 6 months. Cognitive improvements were demonstrated by using a comprehensive cognitive battery. About 52.5% of all treated patients improved in cognition, 30% have shown no change, and 17.5% decreased in cognition. The authors mentioned a strong effect in cognition because of the small cohort. Therefore, they recommend the use of a comprehensive neuropsychological test battery to assess cognitive functions in MS, relating to the MACFIMS (56). Mattioli et al. (57) found improvements in memory and speed processing tasks after 2 years of treatment. In contrast, an addition to the data in the literature, the present study focused only on different aspect of attention but confirm presented results until now.

However, some limiting factors of the present study must be acknowledged. At first, the sample size in both observational periods is still small. Limiting factor of all described results from the literature, as well as from the present results, is the lack of control groups. From ethical point of view, there are many barriers for controlled observational studies to assess the effect of immunomodulatory treatments in patients with MS. To use a placebo or other drug, in comparison to a treatment group, have to be exactly examined ethically.

Furthermore, the comparison between NTZ and other immunomodulatory treatments is very difficult because of the different activity spectrum. Also, practice effects cannot be excluded. In the present study, the time difference between the measurement points is relatively long. Before each attention assessment, several practice trials were conducted to minimize practice effects. A recommendation for controlling the practice effect is an optimal test selection and timing of testing with a repetitive cognitive testing after a longer time periods, e.g., 6 and 12 months (58). A repetitive testing in healthy adults with the subtests alertness and flexibility (TAP) after 6 and 12 months was not significantly improved (58). Therefore, the present data can be evaluated as improvement. The results on the depressed subgroup may be affected by the regression to the mean phenomenon.

In summary, the results of this retrospective open label observational study show a positive effect of NTZ on different aspects of attention and depression in patients with highly active RRMS after 1 year of treatment. The effects were stable also in a subgroup of patients after 2 years of treatment. No effect was detected in fatigue most likely because of the small sample size or the different assessment tools. Despite, fatigue patients improved in information processing speed, divided attention, and cognitive flexibility as well as in the degree of depression. Treatment with NTZ over longer periods may stabilize or improve different aspects of cognition and mood. The observed changes were clinically relevant. Patients reflected more balance, a better mood, lower sadness, a more restful sleep and more power for daily activities, and hobbies. They were more efficient in daily life, showed a better participation in social life, and were able to work again in their profession or a mini-job. The presented results confirm and expand previous published data especially for longer treatment periods. For the clinical practice, a regularly assessment of cognition, mood, and fatigue during the treatment period with NTZ is recommended for detecting improvement as well as regarding the PML risk. For a better comparison between study results, a uniform assessment procedure for the cognitive testing, while treatment with NTZ, and the evaluation of fatigue and depression are needed. Until now for the evaluation of fatigue, the FSMC is recommended (55).

## Conflict of Interest Statement

With respect to the study, all authors report no conflict of interest. Dr. Wolfgang Köhler received honoraria and research support from Bayer HealthCare, Biogen Idec, Genzyme, Merck Serono, Novartis, and Teva. Dr. Jürgen H. Faiss received honoraria and research support from Bayer Health Care, BiogenIdedc, Merck Serono, Novartis, TEVA, and Böhringer Ingelheim.
